# Impacts of WNT1-inducible signaling pathway protein 1 polymorphism on hepatocellular carcinoma development

**DOI:** 10.1371/journal.pone.0198967

**Published:** 2018-06-11

**Authors:** Chih-Tien Chen, Hsiang-Lin Lee, Hui-Ling Chiou, Chia-Hsuan Chou, Po-Hui Wang, Shun-Fa Yang, Ying-Erh Chou

**Affiliations:** 1 Institute of Medicine, Chung Shan Medical University, Taichung, Taiwan; 2 Department of Surgery, Taichung Veterans General Hospital, Taichung, Taiwan; 3 School of Medicine, Chung Shan Medical University, Taichung, Taiwan; 4 Department of Surgery, Chung Shan Medical University Hospital, Taichung, Taiwan; 5 School of Medical Laboratory and Biotechnology, Chung Shan Medical University, Taichung, Taiwan; 6 Department of Clinical Laboratory, Chung Shan Medical University Hospital, Taichung, Taiwan; 7 Department of Obstetrics and Gynecology, Chung Shan Medical University Hospital, Taichung, Taiwan; 8 Department of Medical Research, Chung Shan Medical University Hospital, Taichung, Taiwan; Medizinische Fakultat der RWTH Aachen, GERMANY

## Abstract

**Background:**

WNT1-inducible signaling pathway protein 1 (WISP1) is a member of CCN protein family and a downstream target of β-catenin. Aberrant WISP1 expression is associated with carcinogenesis. In the current study, we focused on examining WISP1 single nucleotide polymorphisms (SNPs) to elucidate hepatocellular carcinoma (HCC) clinicopathologic characteristics.

**Methodology/Principal findings:**

The WISP1 SNPs rs2977530, rs2977537, rs2929973, rs2929970, rs62514004, and rs16893344 were analyzed by real-time polymerase chain reaction in 332 patients with HCC and 664 cancer-free controls.

**Results:**

The patients with higher frequencies of WISP1 rs62514004 (AG + GG) and rs16893344 (CT + TT) variants revealed a lower risk to reach a later clinical stage compared with their wild-type carriers. Furthermore, individuals who carried WISP1 rs62514004 and rs16893344 haplotype G-T showed a greater synergistic effect combined with alcohol drinking on HCC development (AOR = 26.590, 95% CI = 9.780–72.295).

**Conclusions:**

Our results demonstrated that the HCC patients with WISP1 SNPs are associated with HCC development, and WISP1 SNPs may serve as markers or therapeutic targets for HCC.

## Introduction

Hepatocellular carcinoma (HCC) is a deadly cancer; it ranks the second leading cause of male cancer deaths in developing countries and the third most common cause of cancer mortality worldwide [1, 2]. HCC is the first and second leading cause of cancer-related mortality in males and females in Taiwan, respectively, with a crude mortality rate of approximately 30.21 per 100,000 person-years [3–5]. Although options such as ablation therapy, surgical resection, systemic chemotherapy, and transplantation have been developed to treat HCC, the prognosis of HCC remains poor, and the average survival time is 6 to 20 months for untreated HCC patients [6–9]. Moreover, 40%–80% of patients with HCC experience recurrence and metastasis within 5 years after receiving the treatment for HCC [8, 10, 11].

The WNT1-inducible signaling pathway protein 1 (WISP1) is a cysteine-rich protein that belongs to the Cyr61, CTGF, Nov family of matricellular proteins, and it is also known as CCN4 or Elm1 [12, 13]. The “CCN” is the initialism of its first 3 family member. They are connective tissue growth factor, cysteine-rich 61 (CYR61), and nephroblastoma overexpressed (NOV) [13]. The CCN proteins have been suggested to play crucial role in stimulating tumorigenesis in various cancers [14–16]. Aberrant expression of CCN proteins has been observed in some diseases and cancers [13, 17]. WISP1 has been discovered as a downstream target of WNT1, and the transcription factor β-catenin plays the role of mediator in regulating the transcriptional regulation and chromatin interactions to induce tumorigenesis [18, 19]. The expression of WISP1 in different cancers remains ambiguous. WISP1 has been observed to be strongly expressed in human breast and colon cancers and is therefore associated with enhancing tumor growth [18, 20, 21]. It was suggested that WISP1 acts as an oncoprotein in non-small-cell lung cancer (NSCLC), because WISP1 had been overexpressed in NSCLC samples when compared with normal lung tissue [22]. However, it was hypothesized that HCC progression might be enhanced by NOV and suppressed by CYR61 and WISP1 [23]. Although the exact role and mechanisms of WISP1 in cancer remain uncertain, the aberrant expression of WISP1 in numerous cancers implies that it has a role in tumor progression and regulation.

Single nucleotide polymorphisms (SNPs) are one of the most common types of genetic variants in the DNA sequence. Previous studies have discussed the potential roles of WISP1 SNPs in tumors and diseases [24–29]; however, the effects of WISP1 SNPs on HCC development and regulation have not been thoroughly investigated. Our previous study identified that the WISP1 SNPs rs2929970 and rs16893344 are involved in the regulation of oral squamous cell carcinoma (OSCC) [30]. To determine their contribution to HCC clinicopathologic characteristics, we investigated the following 6 WISP1 SNPs; rs2977530, rs2977537, rs2929973, rs2929970, rs62514004, and rs16893344.

## Materials and methods

### Study subjects

From 2007 to 2015, we recruited 332 HCC patients (238 men and 94 women; mean age = 62.5 ± 11.5 years) at Chung Shan Medical University Hospital in Taichung, Taiwan for the case group. They were diagnosed with HCC, according to the characteristic criteria of national guidelines for HCC, such as liver injury diagnosed by either histology or cytology irrespective of a-fetoprotein (AFP) titer where imaging data showed either one of following three cases: (a) one or more liver masses more than or equal to 2 cm in diameter via both computed tomography (CT) and magnetic resonance imaging (MRI); (b) one imaging data with early enhancement and a high level of AFP more than or equal to 400 ng/mL; and (C) one imaging data with early arterial phase contrast enhancement plus early venous phase contrast washout regardless of AFP level. HCC patients were staged clinically at the time of diagnosis according to the TNM staging system of the American Joint Committee on Cancer (AJCC) [31]. Liver cirrhosis was diagnosed by liver biopsy, abdominal sonography, or biochemical evidence of liver parenchymal damage with endoscopic esophageal or gastric varices. During the same study period, 664 (505 men and 159 women; mean age = 55.7 ± 9.5 years) ethnically matched individuals who have neither diagnosed with HCC nor self-reported history of cancer of any sites were enrolled as the controls. To acquire the information on exposure to cigarette smoking and alcohol drinking, we administered a questionnaire for both groups. The medical information of the HCC patients including primary tumor size, TNM clinical staging, distant metastasis vascular invasion, lymph node involvement, Child-Pugh grade, HBsAg and liver cirrhosis was collected from their medical records. Before commencing the study, the approval from the Institutional Review Board of Chung Shan Medical University Hospital and informed written consent from each individual was obtained.

### Sample preparation and DNA extraction

To acquire the genomic DNA, the whole blood specimens collected from HCC patients and normal controls were placed in EDTA containing tubes and were immediately centrifuged 3000 rpm, 10 minutes. DNA extraction was performed to the buffy coats extracted from the whole blood specimens by using a QIAamp DNA blood mini kits, the detail protocols was as described in our previous study [32]. Extracted DNA was dissolved in Tris-EDTA buffer and provided as the template in the following polymerase chain reactions (PCRs).

### Selection of WISP1 polymorphisms

A total of six SNPs in WISP1 were selected from the International HapMap Project data for the current study. We included the SNP rs62514004 in the promoter region. Three SNPs (rs16893344, rs2977530, and rs2977537) which locate in the intron of WISP1, and rs2929970, rs2929973 located in the 3’UTR of WISP1 were selected in the study. Previous study has demonstrated that the WSIP1 rs16893344, rs2977530, rs2977537, and rs62514004 were related to susceptibility of lung cancer [32]. Furthermore, the WISP1 rs62514004 polymorphism has been found significantly associated with platinum-based chemotherapy response in lung cancer patients [32]. The rs2929970 and rs2929973 were selected in this study because these WISP1 SNPs were reported to be associated with multiple diseases such as impaired lung function in asthma and colorectal cancer [33, 34].

### WISP1 SNPs genotyping

To assess the allelic discrimination for the WISP1 rs2977530 (assay IDs: C__16182617_10), rs2977537 (assay IDs: C__16182628_10), and rs2929973 (assay IDs: C__16166873_10), rs2929970 (assay IDs: C__ 9086661_10), rs62514004 (assay IDs: C__88938802_10) and rs16893344 (assay IDs: C__34575402_10) SNP, TaqMan assay was performed with an ABI StepOnePlus^™^ Real-Time PCR System. The data were analyzed and evaluated with SDS version 3.0 software (Applied Biosystems, Foster City, CA, USA).

### Statistical analysis

We adopted Fisher’s exact test and Mann—Whitney U-test to evaluate the differences between the controls and patients with HCC, such as age, gender, alcohol drinking, cigarette smoking, HBsAg, tumor stage, tumor T status, lymph node status, liver cirrhosis, metastasis, Child-Pugh grade. Multiple logistic regression models were used to estimate the odds ratio and 95% CIs of the association between the genotype frequencies and the clinical pathological characteristics and HCC risk. WISP1 haplotype blocks were well-defined by using the default setting of Haploview software (http://sourceforge.net/projects/haploview/) [35]. We evaluated the common haplotypes by PHASE version 2.1. *p* < 0.05 was considered significant. The data of the current study were analyzed on SAS statistical software (Version 9.1, 2005; SAS Institute, Cary, NC).

## Results

Table 1 lists the demographic characteristics of the sample specimens. We observed that 15.5% (103/664) of controls and 37.3% (124/332) of HCC patients drank alcohol (*p* < 0.001). The genotyping and allele frequency of WISP1 genetic polymorphisms between HCC and normal controls are shown in Table 2. In both the controls and patients with HCC, the WISP1 rs2977530, rs2977537, rs2929973, rs2929970, rs62514004, and rs16893344 genetic polymorphisms were most frequent when heterozygous for AG, heterozygous for AG, heterozygous for TG, heterozygous for AG, homozygous for AA, and homozygous for CC, respectively. After adjusting for age, gender, cigarette smoking, and alcohol drinking, no significant differences were observed between HCC patients with the rs2977537, rs2929973, rs2929970, rs62514004, and rs16893344 WISP1 SNPs and those with the wild-type (WT) gene. However, significant differences were observed between the HCC patients with the rs2977530 WISP1 SNP and the controls, with an adjusted odds ratio (AOR) (95% confidence interval (CI)) for AG + GG of 1.380 (1.001–1.903) (Table 2).

**Table 1:** The distributions of demographical characteristics in 664 controls and 332 patients with HCC.

Variable	Controls (N = 664)	Patients (N = 332)	*p* value
Age (yrs)	Mean ± S.D.	Mean ± S.D.	
	55.7 ± 9.5	62.5 ± 11.5	*p* < 0.001*
Gender			
Male	505 (76.0%)	238 (71.7%)	
Female	159 (24.0%)	94 (28.3%)	*p* = 0.136
Cigarette smoking			
No	385 (58.0%)	197 (59.3%)	
Yes	279 (42.0%)	135 (40.7%)	*p* = 0.683
Alcohol drinking			
No	561 (84.5%)	208 (62.7%)	
Hazardous drinking	103 (15.5%)	124 (37.3%)	*p* < 0.001*
HBsAg			
Negative		193 (58.1%)	
Positive		139 (41.9%)	
Stage			
I+II		225 (67.8%)	
III+IV		107 (32.2%)	
Tumor T status			
T1+T2		224 (67.5%)	
T3+T4		108 (32.5%)	
Lymph node status			
N0		323 (97.3%)	
N1+N2+N3		9 (2.7%)	
Metastasis			
M0		315 (94.9%)	
M1		17 (5.1%)	
Child-Pugh grade			
0 or A		257 (77.4%)	
B or C		75 (22.6%)	
Liver cirrhosis			
Negative		56 (16.9%)	
Positive		276 (83.1%)	
α-Fetoprotein (ng/mL)		2803.8 ± 14038.3	
AST (IU/L)		131.5 ± 290.7	
ALT (IU/L)		113.1 ± 228.1	
AST/ALT ratio		1.4 ± 1.5	

Mann-Whitney U test or Fisher’s exact test was used between healthy controls and patients with HCC.

* *p* value < 0.05 as statistically significant.

**Table 2:** Genotyping and allele frequency of *WISP1* single nucleotide polymorphism (SNP) in HCC and normal controls.

Variable	Controls (N = 664) (%)	Patients (N = 332) (%)	Univariate ModelsOR (95% CI)	Multivariable Model 1AOR (95% CI)^a^	Multivariable Model 2AOR (95% CI)^b^
**rs2977530**					
AA	219 (33.0%)	84 (25.3%)	1.000 (reference)	1.000 (reference)	1.000 (reference)
AG	301 (45.3%)	169 (50.9%)	1.464 (1.069–2.004)^c^	1.356 (0.963–1.909)	1.493 (0.999–2.231)
GG	144 (21.7%)	79 (23.8%)	1.430 (0.986–2.075)	1.433 (0.955–2.150)	1.661 (1.044–2.642)^f^
AG+GG	445 (67.0%)	248 (74.7%)	1.453 (1.081–1.953)^d^	1.380 (1.001–1.903)^e^	1.566 (1.078–2.276)^g^
**rs2977537**					
AA	175 (26.4%)	86 (25.9%)	1.000 (reference)	1.000 (reference)	1.000 (reference)
AG	327 (49.3%)	168 (50.6%)	1.046 (0.761–1.473)	1.009 (0.713–1.428)	0.839 (0.556–1.265)
GG	162 (24.3%)	78 (23.5%)	0.980 (0.674–1.424)	0.940 (0.626–1.412)	0.754 (0.472–1.204)
AG+GG	489 (73.6%)	246 (74.1%)	1.024 (0.758–1.382)	0.986 (0.711–1.367)	0.783 (0.534–1.148)
**rs2929973**					
TT	278 (41.9%)	138 (41.6%)	1.000 (reference)	1.000 (reference)	1.000 (reference)
TG	308 (46.4%)	158 (47.6%)	1.033 (0.781–1.367)	1.005 (0.740–1.365)	0.900 (0.492–1.647)
GG	78 (11.7%)	36 (10.8%)	0.930 (0.596–1.450)	0.809 (0.498–1.314)	1.220 (0.420–3.537)
TG+GG	386 (58.1%)	194 (58.4%)	1.012 (0.775–1.323)	0.963 (0.719–1.290)	0.876 (0.483–1.590)
**rs2929970**					
AA	239 (36.0%)	118 (35.5%)	1.000 (reference)	1.000 (reference)	1.000 (reference)
AG	324 (48.8%)	169 (50.9%)	1.056 (0.792–1.410)	1.061 (0.774–1.456)	1.146 (0.620–2.119)
GG	101 (15.2%)	45 (13.6%)	0.902 (0.596–1.366)	0.763 (0.485–1.201)	0.666 (0.243–1.822)
AG+GG	425 (64.0%)	214 (64.5%)	1.020 (0.775–1.343)	0.985 (0.729–1.330)	1.103 (0.598–2.035)
**rs62514004**					
AA	506 (76.2%)	248 (74.7%)	1.000 (reference)	1.000 (reference)	1.000 (reference)
AG	145 (21.8%)	77 (23.2%)	1.083 (0.790–1.485)	1.141 (0.809–1.610)	1.145 (0.797–1.643)
GG	13 (2.0%)	7 (2.1%)	1.099 (0.433–2.788)	1.597 (0.559–4.560)	1.198 (0.368–3.895)
AG+GG	158 (23.8%)	84 (25.3%)	1.085 (0.799–1.472)	1.171 (0.839–1.634)	1.182 (0.833–1.679)
**rs16893344**					
CC	486 (73.1%)	233 (70.2%)	1.000 (reference)	1.000 (reference)	1.000 (reference)
CT	165 (24.9%)	87 (26.2%)	1.100 (0.812–1.489)	0.976 (0.698–1.363)	0.957 (0.674–1.361)
TT	13 (2.0%)	12 (3.6%)	1.925 (0.865–4.285)	1.930 (0.812–4.590)	1.725 (0.676–4.401)
CT+TT	178 (26.9%)	99 (29.8%)	1.160 (0.867–1.552)	1.042 (0.756–1.437)	1.003 (0.715–1.408)

^a^ Multivariable model 1 controlled for age, gender, cigarette smoking, and alcohol drinking.

^b^ Multivariable model 2 controlled for age, gender, cigarette smoking, alcohol drinking, rs2977530, rs2977537, rs2929970, rs62514004, and rs16893344.

^c^ *p* = 0.018.

^d^ *p* = 0.013.

^e^ *p* = 0.049.

^f^ *p* = 0.032.

^g^ *p* = 0.019.

Distribution frequencies of clinical statuses and WISP1 genotypes in patients with HCC were estimated to understand the effect of the WISP1 SNPs on clinical stage, tumor size, vascular invasion, lymph node metastasis, distant metastasis, Child—Pugh grade, HBsAg and liver cirrhosis. The rs2977530, rs2977537, rs2929973, and rs2929970 SNPs showed no significant association with the clinicopathologic statuses. Of the 332 patients with HCC, those carrying the rs62514004 SNP were at lower risks of greater tumor size (OR = 0.438, 95% CI = 0.242–0.791, *p* = 0.006) and reaching a later clinical stage (OR = 0.333, 95% CI = 0.178–0.625, *p* < 0.001) than did those carrying the rs62514004 WT gene; no difference was observed in vascular invasion, lymph node metastasis, distant metastasis, Child—Pugh grade, HBsAg or liver cirrhosis (Table 3). Similar results were observed in patients with HCC carrying the rs16893344 gene. Patients with HCC carrying rs16893344 also exhibited lower risks of greater tumor size (OR = 0.488, 95% CI = 0.284–0.841) and reaching a later clinical stage (OR = 0.425, 95% CI = 0.243–0.742). Again, no difference was observed in other clinical statuses (Table 4).

**Table 3:** Odds ratio (OR) and 95% confidence interval (CI) of clinical status and *WISP1* rs62514004 genotypic frequencies in 332 HCC patients.

Variable	Genotypic frequencies			
	AA (N = 248)	AG+GG (N = 84)	OR (95% CI)	AOR (95% CI) ^a^
Clinical Stage				
Stage I/II	155 (62.5%)	70 (83.3%)	1.00	1.00
Stage III/IV	93 (37.5%)	14 (16.7%)	0.333 (0.178–0.625)^b^	0.344 (0.182–0.651)^d^
Tumor size				
≦ T2	157 (63.3%)	67 (79.8%)	1.00	1.00
> T2	91 (26.7%)	17 (20.2%)	0.438 (0.242–0.791)^c^	0.465 (0.255–0.849)^e^
Lymph node metastasis				
No	239 (96.4%)	84 (100.0%)	1.00	1.00
Yes	9 (3.6%)	0 (0.0%)	-	-
Distant metastasis				
No	233 (94.0%)	82 (97.6%)	1.00	1.00
Yes	15 (6.0%)	2 (2.4%)	0.379 (0.085–1.693)	0.328 (0.071–1.512)
Vascular invasion				
No	203 (81.9%)	72 (85.7%)	1.00	1.00
Yes	45 (18.1%)	12 (14.3%)	0.752 (0.377–1.501)	0.719 (0.356–1.453)
Child-Pugh grade				
A	194 (78.2%)	63 (75.0%)	1.00	1.00
B or C	54 (21.8%)	21 (25.0%)	1.198 (0.671–2.136)	1.144 (0.633–2.066)
HBsAg				
Negative	145 (58.5%)	48 (57.1%)	1.00	1.00
Positive	103 (41.5%)	36 (42.9%)	1.056 (0.640–1.742)	1.105 (0.643–1.899)
Liver cirrhosis				
Negative	44 (17.7%)	12 (14.3%)	1.00	1.00
Positive	204 (82.3%)	72 (85.7%)	1.294 (0.647–2.586)	1.186 (0.582–2.416)

The ORs with analyzed by their 95% CIs were estimated by logistic regression models.

> T2: multiple tumor more than 5 cm or tumor involving a major branch of the portal or hepatic vein(s)

^a^ Adjusted for the effects of age, gender, cigarette smoking, and alcohol drinking.

^b^ *p* < 0.001;

^c^ *p* = 0.006;

^d^ *p* = 0.001;

^e^ *p* = 0.013.

**Table 4:** Odds ratio (OR) and 95% confidence interval (CI) of clinical status and *WISP1* rs16893344 genotypic frequencies in 332 HCC patients.

Variable	Genotypic frequencies			
	CC (N = 233)	CT+TT (N = 99)	OR (95% CI)	AOR (95% CI) ^a^
Clinical Stage				
Stage I/II	146 (62.7%)	79 (79.8%)	1.00	1.00
Stage III/IV	87 (37.3%)	20 (20.2%)	0.425 (0.243–0.742)^b^	0.439 (0.249–0.774)^d^
Tumor size				
≦ T2	147 (63.1%)	77 (77.8%)	1.00	1.00
> T2	86 (36.9%)	22 (22.2%)	0.488 (0.284–0.841)^c^	0.511 (0.294–0.890)^e^
Lymph node metastasis				
No	226 (97.0%)	97 (98.0%)	1.00	1.00
Yes	7 (3.0%)	2 (2.0%)	0.666 (0.136–3.262)	0.615 (0.121–3.126)
Distant metastasis				
No	218 (93.6%)	97 (98.0%)	1.00	1.00
Yes	15 (6.4%)	2 (2.0%)	0.300 (0.067–1.336)	0.258 (0.056–1.183)
Vascular invasion				
No	191 (82.0%)	84 (84.9%)	1.00	1.00
Yes	42 (18.0%)	15 (15.1%)	0.812 (0.427–1.545)	0.791 (0.410–1.524)
Child-Pugh grade				
A	184 (78.0%)	73 (73.7%)	1.00	1.00
B or C	49 (22.0%)	26 (26.3%)	1.337 (0.774–2.312)	1.267 (0.724–2.216)
HBsAg				
Negative	138 (59.2%)	55 (55.6%)	1.00	1.00
Positive	95 (40.8%)	44 (44.4%)	1.162 (0.723–1.868)	1.344 (0.801–2.256)
Liver cirrhosis				
Negative	42 (18.0%)	14 (14.1%)	1.00	1.00
Positive	191 (82.0%)	85 (85.9%)	1.335 (0.692–2.574)	1.200 (0.611–2.356)

The ORs with analyzed by their 95% CIs were estimated by logistic regression models.

> T2: multiple tumor more than 5 cm or tumor involving a major branch of the portal or hepatic vein(s)

^a^ Adjusted for the effects of age, gender, cigarette smoking, and alcohol drinking.

^b^ *p* = 0.003;

^c^ *p* = 0.010;

^d^ *p* = 0.005;

^e^ *p* = 0.018.

We also analyzed the levels of AFP, AST, and ALT, which are common clinical pathological markers of HCC associated with WISP1 genotypic frequencies, to observe their relationship with the progress of the clinical status of patients with HCC. Table 5 exhibits the associations of WISP1 genotypic frequencies with HCC laboratory status. No significant association was observed with the rs2977530, rs2977537, rs2929973, rs2929970, rs62514004, or rs16893344 gene polymorphisms.

**Table 5:** Association of *WISP1* genotypic frequencies with the HCC laboratory findings.

Characteristic	α-Fetoprotein ^a^ (ng/mL)	AST ^a^ (IU/L)	ALT ^a^ (IU/L)	AST/ALT ratio ^a^
**rs2977530**				
AA	2536.9 ± 1783.9	117.8 ± 35.9	119.0 ± 30.2	1.3 ± 0.1
AG+GG	2894.1 ± 838.2	136.1 ± 17.6	111.1 ± 13.3	1.5 ± 0.1
*p* value	0.857	0.648	0.813	0.086
*p* value^b^	0.839	0.618	0.786	0.188
**rs2977537**				
AA	2325.8 ± 1740.9	148.2 ± 42.4	146.1 ± 39.4	1.26 ± 0.1
AG+GG	2970.9 ± 845.2	125.7 ± 15.7	101.6 ± 9.8	1.51 ± 0.1
*p* value	0.739	0.620	0.276	0.064
*p* value^b^	0.712	0.537	0.119	0.174
**rs2929973**				
TT	3296.4 ± 1574.8	133.7 ± 29.1	131.8 ± 26.7	1.41 ± 0.2
TG+GG	2453.3 ± 907.0	129.9 ± 17.9	99.8 ± 9.9	1.47 ± 0.1
*p* value	0.604	0.913	0.263	0.711
*p* value^b^	0.588	0.908	0.207	0.687
**rs2929970**				
AA	3783.4 ± 1572.8	117.1 ± 23.2	121.7 ± 24.6	1.3 ± 0.1
AG+GG	2263.5 ± 823.4	139.4 ± 21.2	108.4 ± 13.9	1.5 ± 0.1
*p* value	0.393	0.477	0.639	0.131
*p* value^b^	0.342	0.502	0.611	0.171
**rs62514004**				
AA	2734.9 ± 867.1	136.5 ± 19.6	110.6 ± 13.2	1.5 ± 0.1
AG+GG	3007.0 ± 1658.1	116.6 ± 25.0	120.5 ± 30.8	1.4 ± 0.1
*p* value	0.878	0.532	0.767	0.326
*p* value^b^	0.877	0.588	0.730	0.496
**rs16893344**				
CC	2858.1 ± 920.4	133.2 ± 19.7	109.7 ± 13.4	1.5 ± 0.1
CT+TT	2675.9 ± 1415.5	127.6 ± 26.7	121.2 ± 27.8	1.3 ± 0.1
*p* value	0.914	0.872	0.711	0.267
*p* value^b^	0.913	0.872	0.675	0.406

Mann-Whitney U test was used between two groups.

^a^ Mean ± S.E.

^b^ Adjusted age, gender, cigarette smoking, and alcohol drinking.

**Fig 1:**
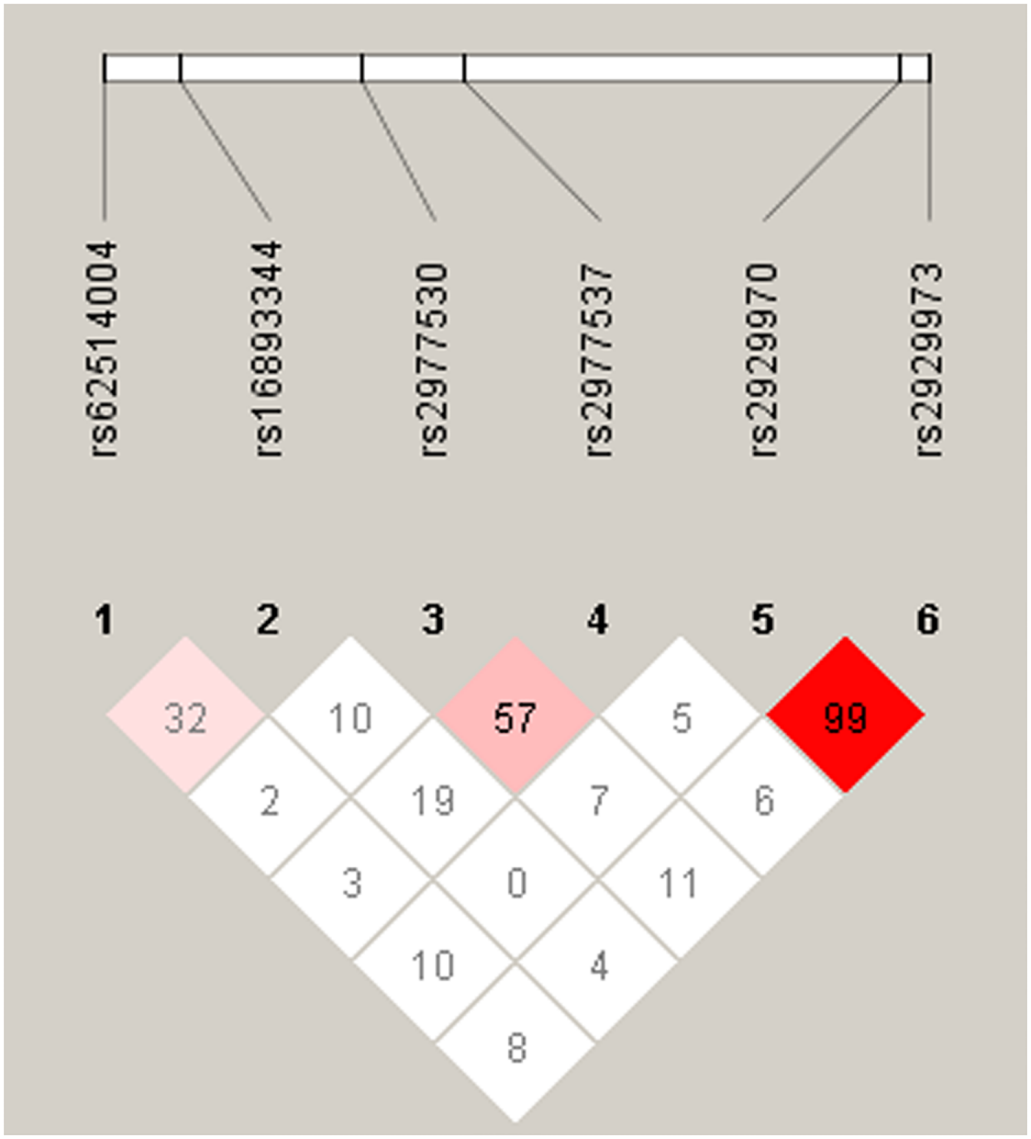
The pairwise linkage disequilibrium patterns of WISP1 gene. Block is pairwise *D*’ plots and haplotype blocks obtained from HAPLOVIEW.

To analyze the common haplotypes, we used Haploview software and the PHASE program. As shown in Fig 1 and Table 6, compared with the A-C reference group (WISP1 rs62514004/rs16893344), carriers with A-T or G-C had significant 0.339-fold (95% CI 0.219–0.524) and 0.190-fold (95% CI 0.100–0.363) lower risks of HCC. However, carriers with G-T had a significant 3.321-fold (95% CI 2.196–5.020) increased risk of HCC (Table 6).

**Table 6:** Frequencies of *WISP1* haplotypes in HCC patients and control subjects.

Haplotype block		Controls n = 1328	Patients n = 664	AOR (95% CI)^a^
rs62514004 A/G	rs16893344 C/T			
A	C	1011 (76.1%)	542 (81.6%)	1.000 (reference)
A	T	146 (11.0%)	31 (4.7%)	0.339 (0.219–0.524)^b^
G	C	126 (9.5%)	11 (1.7%)	0.190 (0.100–0.363)^b^
G	T	45 (3.4%)	80 (12.0%)	3.321 (2.196–5.020)^b^

^a^ Adjusted for the effects of age, gender, cigarette smoking, and alcohol drinking.

^b^ *p* < 0.001

Alcohol drinking is an established environmental risk factor for HCC development. To determine whether the WISP1 (rs62514004/rs16893344) G-T haplotypes were associated with environmental risk factors for HCC development, the combined effects of WISP1 haplotypes (rs62514004/rs16893344) and alcohol drinking were observed in patients with HCC. Compared with the reference group and nondrinkers carrying haplotypes A-C, A-T, and G-C, those carrying haplotype G-T had a significant 3.605-fold (95% CI 2.280–5.699) increased risk of HCC. For all alcohol drinkers (both patients with HCC and controls), carriers with the A-C, A-T, and G-C haplotypes had a significant 4.682-fold (95% CI 3.558–6.161) increased risks of HCC compared with the reference group. Furthermore, the alcohol drinkers with the WISP1 G-T haplotype had greatly increased risks of HCC, with a significant 26.590-fold (95% CI 9.780–72.295) increase compared with the reference group.

## Discussion

In this study, we investigated WISP1 SNPs and their relationships with HCC. Alcohol is a common risk factor for HCC, and chronic ethanol (EtOH) consumption has been suggested to activate the Wnt/β-catenin signaling pathway, leading to increased hepatocyte proliferation, thus promoting tumorigenesis following an initiating insult in the liver [36]. EtOH metabolism by alcohol dehydrogenase and cytochrome P450 (CYP) CYP2E1 produces the reactive metabolite acetaldehyde, leading to reduced DNA methylation and resulting in the disruption of one-carbon metabolism. This may contribute to tumorigenesis [37]. Our data revealed that the WISP1 SNP rs2977530 (AG + GG) was associated with HCC development (Table 2). Furthermore, we discovered that the WISP1 SNPs rs62514004 (AG + GG) and rs16893344 (CT + TT) were correlated with lower risks of greater tumor size and reaching a later clinical stage of HCC (Tables 3 and 4).

The WISP1 SNPs rs16893344 and rs2977530 were located in introns, and rs62514004 was situated in the promoter regions of the *WISP1* gene [29]. Chen et al. indicated that WISP1 SNPs rs2977530, rs62514004, and rs16893344 were related to lung cancer susceptibility, and that rs16893344 and rs62514004 were significantly associated with platinum-based chemotherapy response. The T allele of WISP1 rs16893344 and the A alleles of rs2977530 and rs62514004 are correlated with increased risk of lung cancer [29]. Our study, however, revealed that the WISP1 rs2977530 G allele was associated with HCC, whereas the WISP1 SNPs rs62514004 and rs16893344 were not (Table 2). Notably, we found that the WISP1 rs62514004 (AG + GG) and rs16893344 (CT + TT) genetic variants were associated with lower risks of greater tumor size and reaching a later clinical stage (Tables 3 and 4). These data suggested that WISP1 rs62514004 represents the same tumorigenesis effect in lung cancer and HCC, because the WT A allele is associated with increased risk of lung cancer, whereas the polymorphic G allele is correlated with lower risks of greater tumor size and reaching a later clinical stage in HCC. The WISP1 SNP rs62514004 may have contributed to WISP1 regulation, because it was located in the promoter region of the *WISP1* gene. By contrast, the effects of rs2977530 and rs16893344 polymorphisms in lung cancer and HCC were opposite. The rs2977530 WT A allele and rs16893344 polymorphic T allele were associated with increased risk of lung cancer [29], whereas the rs2977530 polymorphic G allele was associated with HCC development, and the rs16893344 polymorphic T allele was associated with a lower risk of poorer HCC clinical development (Tables 2 and 4). Although WISP1 rs2977530 and rs16893344 were located in introns, previous studies have indicated that the expression of certain genes may be regulated by their genetic variations [38, 39]. These results demonstrated the variety of WISP1 polymorphisms in different cancers. Such inconsistencies may have been caused by the nature of WISP1 expression in different diseases or the different signaling pathways and mechanisms induced by carcinogen exposure for each cancer. Although WISP1 has been suggested to be overexpressed in many cancers [17], it was indicated that HCC progression may be enhanced by NOV and suppressed by WISP1 and CYR61 [23]. Therefore, the tumor-suppression characteristics exhibited by the WISP1 rs62514004 polymorphic variant G and rs16893344 polymorphic variant T may explain this phenomenon.

To determine whether WISP1 SNPs were correlated with HCC clinical statuses, we examined the association of WISP1 genotypic frequencies with the HCC laboratory findings. No significant differences were found between WISP1 SNPs and HCC laboratory findings, implying that the effect of WISP1 SNP expression on HCC carcinogenesis was limited (Table 5). We evaluated the frequencies of WISP1 haplotypes in both HCC patients and control subjects. Compared with the WISP1 SNP rs62514004 and rs16893344 WT A-C haplotype control group, we found that the WISP1 SNP rs62514004 and rs16893344 G-T haplotype carried a greater risk for HCC, whereas the A-T and G-C haplotypes represented lower risk of HCC (Table 6). Notably, the WISP1 rs62514004 and rs16893344 G-T haplotype combined with drinking alcohol dramatically increased the risk of HCC (Table 7). These results indicated that alcohol drinking would not only abolish the tumor-suppressing activity of WISP1 haplotypes A-T and G-C, but also generate increased risk of HCC (Tables 6 and 7). Even carriers of other haplotypes, including A-C, A-T, and G-C, who drank alcohol exhibited a 4.682-fold to 3.605-fold higher risk of HCC compared with nondrinking G-T haplotype carriers (Table 7). These data support that alcohol promotes HCC tumorigenesis and suggest a synergistic effect in alcohol drinkers with the WISP1 G-T haplotype, leading to poorer HCC prognoses. However, the interactions between WISP1 SNPs and alcohol drinking remained mostly uninvestigated. Previous studies have suggested that hepatic retinoid storage loss occurs in HCC development and the progression of alcoholic liver disease [40, 41]. In animal models of alcoholic liver disease and HCC, chronic EtOH feeding has increased hepatocyte proliferation [42–47]. Connections between retinoid depletion, upregulation of Wnt signaling, HCC tumor promotion, and increased hepatocyte proliferation were suggested by Mercer et al., and the *WISP1* gene exhibited the highest relative expression among Wnt signaling components and β-catenin targets, with a 3.3-fold change following EtOH consumption in rats [36]. Therefore, retinoid acid depletion might develop in response to the synergistic effect between alcohol consumption and the WISP1 G-T haplotype. Retinoic acid has been considered as treatment for lung, prostate, breast, ovarian, bladder, oral, and skin cancers [48]. Lower levels of retinoic acid were observed in patients with lung cancer, and retinol deficiency or retinol metabolism impairment has been suggested as playing a role in cancer [49]. In rat models, low vitamin A was suggested to increase susceptibility to the development of cigarette smoke-induced lung emphysema, and vitamin A depletion induced by cigarette smoke was associated with increased expression of lung cancer-related markers [50, 51]. Although the exact interactions of WISP1 SNPs and alcohol consumption in HCC remain unclear, retinoic acid depletion may play an essential role in WISP1 SNPs expression when combined with alcohol drinking in HCC. A previous case-control study involving patients with HCC in the United States indicated that heavy alcohol consumption (≥80 g daily) was a primary factor responsible for one-third of the reported HCC cases [52]. Of the HCC patients in our study group, 37.3% consumed alcohol and 40.7% smoked cigarettes. Because both alcohol drinking and cigarette smoking have been associated with retinoic acid depletion [40, 41, 51], our study is limited by our lack of the data regarding hepatic retinoid level and exact levels of alcohol or cigarette consumption in HCC patients. Thus, detailed correlation analysis of the expressions of WISP1 SNPs could not be performed. Further studies are required to identify the exact mechanisms of WISP1 SNPs related to HCC development, especially the synergistic effect of alcohol consumption combined with the WISP1 rs62514004 and rs16893344 G-T haplotype.

**Table 7:** Combined effect of alcohol drinking and *WISP1* haplotypes on HCC development.

Alcohol drinking	*WISP1* haplotype	Controls	Patients	AOR (95% CI)^b^
		n = 1382	n = 664	
Yes	G-T	5 (0.4%)	28 (4.3%)	26.590 (9.780–72.295)^c^
Yes	Others^a^	201 (15.1%)	220 (33.1%)	4.682 (3.558–6.161)^c^
No	G-T	40 (3.0%)	52 (7.8%)	3.605 (2.280–5.699)^c^
No	Others^a^	1082 (81.5%)	364 (54.8%)	1.000 (reference)

^a^ Other haplotypes included A-C, A-T, and G-C.

^b^ Adjusting for the effects of age, gender, and cigarette smoking.

^c^ *p* < 0.001

*p*-values were adjusted for multiple comparisons by applying the Bonferroni correction.

The limitations to our study is that our sample is modest, so larger independent cohort study is required to confirm the result and phenomenon we discovered. Besides, it is not known whether the controls had underlying liver disease or not, or if they had cirrhosis that need to be addressed by future studies. Moreover, there is lack of the underlying etiology/liver disease of HCC, overall survival, progression free survival, disease free survival or Barcelona Clinic Liver Cancer-B (BCLC-B) stadium information, which may be better interpretation of the WISP1 SNPs as a cohort study.

In conclusion, our study first determined the correlation between WISP1 SNPs and HCC. The WISP1 rs2977530 was associated with HCC development, whereas WISP1 rs62514004 and rs16893344 polymorphisms exhibited tumor-suppressing characteristics. Furthermore, we identified a combined effect of alcohol drinking with the WISP1 rs62514004 and rs16893344 G-T haplotype; a synergistic effect with alcohol drinking not only negated the tumor-suppressing activity of WISP1 rs62514004 and rs16893344 polymorphisms but also resulted in a poorer prognosis for HCC development. Our results indicated that EtOH acts as a tumor promoter and may trigger additional targets in the hepatic Wnt/β-catenin signaling system. WISP1 SNPs may serve as potential markers or therapeutic targets for HCC.
